# A Relação Causal entre Microbiota Intestinal e Fibrilação Atrial: Um Estudo de Randomização Mendeliana de Duas Amostras

**DOI:** 10.36660/abc.20240357

**Published:** 2024-11-18

**Authors:** Yuan Zhou, Xuan Wang, Jiongchao Guo, Lei Zhang, Huangsheng Zheng

**Affiliations:** 1 Department of Cardiology The First People’s Hospital of Hefei The Third Affiliated Hospital of Anhui Medical University Hefei China Department of Cardiology, The First People’s Hospital of Hefei, The Third Affiliated Hospital of Anhui Medical University, Hefei – China; 2 Department of Health Medicine The First Affiliated Hospital of Xi’an Jiaotong University Shanxi Province China Department of Health Medicine, The First Affiliated Hospital of Xi’an Jiaotong University, Shanxi Province – China

**Keywords:** Análise da Randomização Mendeliana, Microbioma Gastrointestinal, Fibrilação Atrial, Doenças Cardiovasculares

## Abstract

**Fundamento:**

Estudos anteriores caracterizaram adequadamente a microbiota intestinal (MI) na fibrilação atrial (FA). No entanto, a causalidade precisa entre a MI e a FA permanece obscura.

**Objetivos:**

O presente estudo utilizou dados públicos de estudos de associação genômica ampla para explorar a causalidade entre MI e FA.

**Métodos:**

Na primeira de duas rodadas de análise de randomização mendeliana (RM), as variáveis instrumentais (VIs) incluíram polimorfismos de nucleotídeo único (SNPs) que ficaram abaixo do limite de significância estatística de todo o genoma (5 × 10^-8^). Para chegar a uma conclusão mais abrangente e inclusiva, selecionamos ainda SNPs abaixo do nível de significância de todo o lócus (1 × 10^-5^) como VIs para o segundo grupo. A análise de RM considerou o efeito causal estatisticamente significativo entre MI específica e FA quando p < 0,05. Além disso, na análise de sensibilidade, p > 0,05 não indicou heterogeneidade nem pleiotropia.

**Resultados:**

No limiar de significância de todo o lócus, os resultados demonstraram um impacto causal da MI no risco de FA. O método de ponderação de variância inversa indicou que *Actinobacteria*, *Firmicutes*, *Alloprevotella*, *Bifidobacterium*, *Blautia*, *Eggerthella*, *Howardella*, *Ruminococcaceae UCG004* e *Ruminococcus1* foram negativamente correlacionados com FA, ao passo que *Pasteurellales*, *Pasteurellaceae*, *Oxalobacter*, *Ruminiclostridium5* e *Turicibacter* foram positivamente correlacionados. Além disso, no limiar de significância de todo o genoma, *Actinobacteria*, *Bifidobacteriaceae* e *Bifidobacterium* foram fatores de proteção para o risco de desenvolver FA, ao passo que *Oxalobacteraceae* e *Erysipelatoclostridium* foram fatores de risco para FA. Entretanto, análises de sensibilidade mostraram heterogeneidade ou pleiotropia horizontal nos resultados para *Actinobacteria*, *Howardella*, *Oxalobacter* e *Firmicutes*.

**Conclusões:**

Este estudo fornece evidências da existência de causalidade favorável e desfavorável da MI no risco de FA.

## Graphical abstract

**Figura Central:**
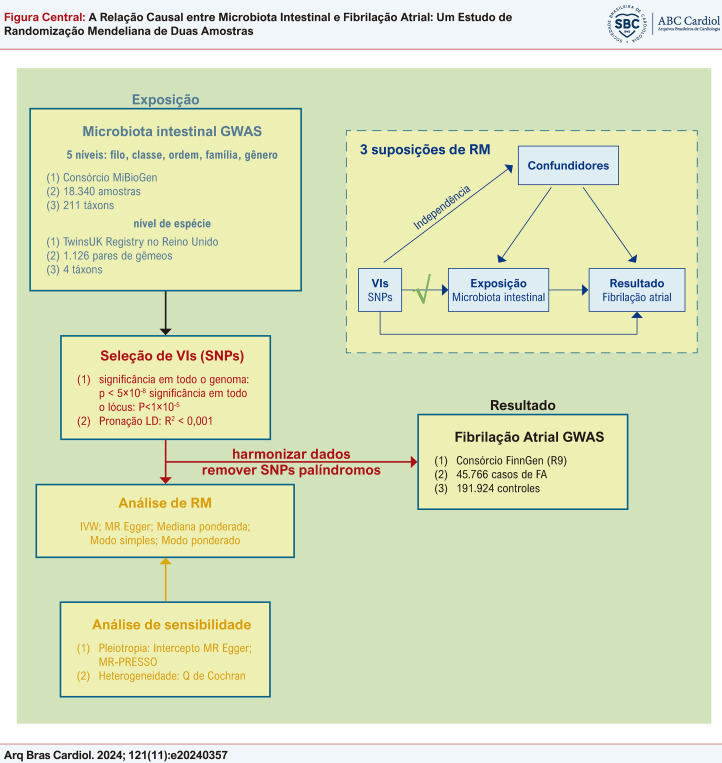
: A Relação Causal entre Microbiota Intestinal e Fibrilação Atrial: Um Estudo de Randomização Mendeliana de Duas Amostras Visão geral do processo de análise de randomização mendeliana (RM) e principais suposições.

## Introdução

A fibrilação atrial (FA) é uma arritmia prevalente e complexa que geralmente se manifesta em indivíduos com mais de 60 anos e atualmente afeta mais de 370 mil indivíduos em todo o mundo.^1^ A FA é caracterizada por interrupções no processo de despolarização dos átrios, podendo resultar em sintomas como palpitações, desconforto no peito, falta de ar e tensão psicológica devido às contrações atriais irregulares.^2^ Complicações mais sérias, incluindo acidentes vasculares cerebrais isquêmicos (infartos cerebrais), insuficiência cardíaca e até morte, também podem ocorrer.^3^ Observações clínicas e pré-clínicas indicam uma combinação de diversos fatores de risco cardiovascular modificáveis que influenciam o início e o avanço da FA, como idade, sexo, doença arterial coronária, insuficiência cardíaca, hipertensão, diabetes mellitus e obesidade.^4^ Necessita-se urgentemente da elucidação adicional da patogênese da FA para melhorar sua prevenção e tratamento.

O intestino humano serve como habitat para diversos microrganismos não patogênicos, conhecidos coletivamente como microbiota intestinal (MI), que é essencial para moldar e facilitar as operações dos sistemas metabólico e imunológico. Estudos clínicos recentes e experimentos de base revelaram que a MI possivelmente influencia as vias de doenças por meio dos metabólitos gerados nos intestinos. Notavelmente, a associação entre MI e doenças cardiovasculares (DCV) ganhou força significativa em pesquisas em andamento. Em um estudo, o conteúdo fecal de doadores humanos hipertensos foi transplantado em camundongos livres de germes e os aumentos diretos subsequentes na pressão arterial puderam ser atribuídos à MI.^5^ Além disso, descobriu-se que a MI contribui para a exacerbação da função cardíaca e progressão da fibrose miocárdica em camundongos com insuficiência cardíaca por meio do metabolismo da colina da dieta em N-óxido de trimetilamina (TMAO).^6^ Embora alguns estudos tenham abordado o perfil diversificado da MI na FA, ainda não está claro se existe uma relação causal entre MI e FA.

A técnica de randomização mendeliana (RM), que envolve a fusão de dados agregados de estudos de associação genômica ampla (GWAS), permite mitigar a influência de fatores de confusão. Portanto, a RM se destaca como uma abordagem predominante para deduzir a existência de uma conexão causal entre exposição e resultado. Variantes genéticas que exibem associações significativas com a exposição são escolhidas como variáveis instrumentais (VIs) para deduzir causalidade. Se a exposição for causal, as VIs que afetam a exposição influenciarão o resultado proporcionalmente.^7^ No estudo atual, foi realizada RM de duas amostras para examinar se existe uma relação causal entre MI e o risco de FA.

## Métodos

### Fontes de dados

Aproveitando dados do consórcio MiBioGen, Kurilshikov et al.^8^ aproveitaram perfis de sequenciamento do gene 16S rRNA e informações de genotipagem de uma coleção de 18.340 amostras para explorar a intrincada interação entre variantes genéticas e a MI. A totalidade dos participantes do consórcio MiBioGen abrangeu populações europeias de 11 países, constituindo um coletivo de 25 coortes distintas. Por meio de um exame das variações nos táxons da MI, o estudo GWAS por fim extraiu 122.110 locais distintos de variação genética originários de 211 táxons em vários níveis (filo, classe, ordem, família, gênero). A partir desse GWAS em larga escala, as VIs correspondentes aos táxons da MI foram derivadas em cinco níveis. Como os dados adquiridos do consórcio MiBioGen não tinham análise no nível de espécie, também obtivemos VIs para táxons da MI no nível de espécie do estudo TwinsUK Registro GWAS.^9^ Goodrich et al. geraram VIs de táxons da MI no nível de espécie a partir de 1.126 pares de gêmeos para dados de sequenciamento de 16S rRNA, definindo, por fim, quatro espécies qualificadas.^9^

As estatísticas resumidas do GWAS para FA foram extraídas da versão mais recente (versão R9) do projeto de pesquisa FinnGen (https://r9.finngen.fi/).^10^ As análises foram realizadas usando 45.766 casos de FA e 191.924 controles após ajustes para idade, sexo, parentesco genético, lote de genotipagem e os primeiros 10 componentes principais separadamente.

Para o desenho de um estudo de RM válido, o tamanho da amostra necessária deve ser calculado. De acordo com a teoria estatística assintótica,^11,12^ o tamanho mínimo da amostra derivado do uso de ferramentas da web disponíveis (https://sb452.shinyapps.io/power/) no nível de significância (< 0,05) e poder (> 80%) dado deve ser 9900. O tamanho da amostra incluído em nosso estudo de pesquisa conseguiu atender aos critérios.

### Seleção de polimorfismo de nucleotídeo único

Para garantir a credibilidade e a precisão das conclusões sobre o nexo causal entre MI e o risco de FA, diversos procedimentos de controle de qualidade foram empregados para escolher meticulosamente as VIs mais confiáveis. Primeiro, os polimorfismos de nucleotídeo único (SNPs) que exibiam associações significativas com a MI foram escolhidos como VIs, com dois limites distintos aplicados para facilitar essa seleção e atingir um escopo mais abrangente de resultados: uma coleção de SNPs abaixo do limite de significância estatística de todo o genoma (5 × 10^-8^), bem como um grupo adicional de SNPs abaixo do nível de significância de todo o lócus (1 × 10^-5^).^13^ Em segundo lugar, foi necessário avaliar a força da correlação entre VIs e exposição. Essas avaliações são normalmente realizadas por meio da estatística F, que foi calculada como $F=R^{2}(n-k-1)/k\left(1-R^{2}\right)$ , onde R^2^ representa a variância de exposição explicada pelos SNPs selecionados, n é o tamanho da amostra e k representa o número de VIs incluídas. Se a estatística F for menor que 10, isso significa uma conexão fraca entre VIs e exposição, então os SNPs que contribuem para essas VIs foram excluídos. Em segundo lugar, o limite para a frequência do alelo menor (MAF) da variante em consideração foi definido em 0,01. Terceiro, os SNPs em alto desequilíbrio de ligação (LD) foram excluídos das VIs; a presença de LD substancial poderia introduzir resultados tendenciosos. Neste estudo, o LD entre os SNPs incluídos foi avaliado pelo processo de aglomeração (R^2^ < 0,001 e distância de aglomeração = 10.000 kb). Quarto, uma faceta fundamental da RM envolve a confirmação de que os efeitos dos SNPs na exposição pertencentes ao mesmo alelo se alinham com os efeitos no resultado. Em conformidade com esse princípio, os SNPs palindrômicos foram excluídos das VIs. Quinto, nos casos em que os SNPs vinculados à exposição não foram identificados no resultado GWAS, SNPs substitutos que apresentaram associações significativas com as variantes pertinentes foram substituídos (r^2^> 0,8).

### As suposições da randomização mendeliana

Para realizar uma análise de RM de duas amostras precisa e padronizada, as três suposições abaixo tiveram que ser atendidas:^14^ (1) as VIs incluídas para utilização devem exibir uma forte associação com os táxons da MI; (2) as VIs incluídas e os fatores de confusão (que influenciam a MI e a FA) devem permanecer independentes uns dos outros; e (3) não deve existir nenhuma pleiotropia horizontal, o que significa que as VIs afetam a FA apenas por meio dos táxons da MI. Nossos resultados foram relatados simultaneamente seguindo as orientações MR-STROBE^15^ (Tabela S1). Este estudo não exigiu consentimento informado ou aprovação ética porque utilizou dados disponíveis publicamente.

### Análise estatística

No presente estudo, empregamos diferentes técnicas de RM, incluindo ponderação de variância inversa (IVW), MR-Egger, mediana ponderada, modo simples e modo ponderado, para verificar a possível influência causal da composição da MI no risco de FA. Neste cenário, o método primário empregado para análise de RM foi o IVW, que funciona essencialmente como uma técnica de meta-análise que se transforma em uma regressão ponderada dos efeitos dos resultados das VIs sobre os efeitos da exposição para produzir uma estimativa abrangente da influência da MI no risco de FA, ao mesmo tempo em que restringe o intercepto a zero. Na ausência de pleiotropia horizontal, o IVW poderia efetivamente contornar a influência de fatores de confusão, permitindo estimativas imparciais.^16^ Além disso, existem vários outros métodos para complementar os resultados do IVW. A regressão MR-Egger pode identificar e contabilizar a pleiotropia, embora essa abordagem frequentemente gere estimativas com precisão limitada.^17^ A abordagem da mediana ponderada fornece estimativas confiáveis considerando a suposição de que um mínimo de 50% das VIs são válidas.^18^ Embora o modo simples possa não possuir o mesmo nível de potência que o IVW, ele oferece resiliência contra os efeitos da multivalência.^19^ Por fim, o modo ponderado é suscetível a variações na seleção da largura de banda para estimativa do modo.^20^

As análises de sensibilidade geralmente foram realizadas em três etapas. A regressão MR-Egger, um método que permite identificar e contabilizar a pleiotropia na análise de RM ao mesmo tempo em que deriva estimativas de efeitos causais e analisa se os resultados são influenciados pela pleiotropia horizontal direcionada, foi inicialmente aplicada para avaliar a possível presença de efeitos de pleiotropia horizontal entre os SNPs incluídos.^17,21^Considerando as limitações de precisão e poder estatístico associadas à regressão MR-Egger, a abordagem de soma residual e valores discrepantes da pleiotropia RM (MR-PRESSO) foi empregada para identificar possíveis valores discrepantes que poderiam indicar viés de pleiotropia e, posteriormente, corrigir os efeitos de pleiotropia horizontal.^22^ Por fim, a estatística Q de Cochran foi empregada para medir o grau de heterogeneidade entre os SNPs selecionados.^23,24^

Todas as análises estatísticas foram executadas usando o software R (versão 4.3.1). O pacote R TwoSampleMR (versão 0.5.7, Stephen Burgess, Chicago, IL, EUA) foi usado para realizar análises de RM de causalidade entre MI e FA. P < 0,05 foi considerado um possível efeito causal estatisticamente significativo entre exposição e desfecho.

## Resultados

### Fontes de dados

A Figura Central ilustra o desenho do estudo e o fluxo específico da análise de RM entre MI e FA. As estatísticas resumidas do GWAS para a MI foram obtidas do consórcio MiBioGen e do consórcio TwinsUK Registry, que contêm 18.340 e 1.126 indivíduos, respectivamente. Além disso, as estatísticas resumidas do GWAS para FA foram obtidas do consórcio FinnGen, que contém 45.766 casos de FA e 191.924 controles. A Tabela 1 mostra os detalhes de três conjuntos de dados.

**Tabela 1:** – Descrição das fontes de dados para microbiota intestinal e fibrilação atrial

Características	Consórcio	Tamanho da amostra	População	Ano	Periódico
Microbiota intestinal	MiBioGen	18.340 indivíduos	Europeia	2021	Nature Genetics
	TwinsUK Registry	1.126 indivíduos	Europeia	2016	Cell Host & Microbe
Fibrilação atrial	FinnGen (R9)	45.766 casos e 191.924 controles	Europeia	2022	Nature

### Seleção das variáveis instrumentais

Após uma rigorosa etapa de controle de qualidade, 2.182 SNPs foram identificados como VIs associados a 207 táxons da MI no nível de significância de todo o lócus (p < 1 × 10^-5^). Esses táxons compreendiam quatro espécies (14 SNPs), nove filos (102 SNPs), 10 classes (109 SNPs), 19 ordens (210 SNPs), 35 famílias (385 SNPs) e 130 gêneros (1.362 SNPs). Cada SNP apresentou validade suficiente (variando de 14,59 a 88,43, todos F > 10; Tabela 2). A Tabela S2 fornece informações abrangentes sobre as VIs.

**Tabela 2:** – Seleção das VIs após controle de qualidade

Taxonomias	p < 1e-05			p < 5e-08		
	Táxons	VIs	Alcance (estatísticas F)	Táxons	VIs	Alcance (estatística F)
Espécie	4	14	22,50-30,84	1	2	30,25-30,84
Filo	9	102	16,97-58,16	1	1	58,16
Classe	10	109	17,61-85,38	1	1	85,38
Ordem	19	210	17,68-85,37	1	1	30,07
Família	35	385	16,91-85,37	5	6	29,81-85,37
Gênero	130	1362	14,59-88,43	11	12	29,35-88,43
Total	207	2182	14,59-88,43	20	23	29,35-88,43

*VIs: variáveis instrumentais.*

Além disso, no limiar de significância estatística de todo o genoma (p < 5 × 10^-8^), apenas 23 SNPs foram identificados como VIs, associados a 20 táxons da MI. Esses táxons compreendiam uma espécie (2 SNPs), um filo (1 SNP), uma classe (1 SNP), uma ordem (1 SNP), cinco famílias (6 SNPs) e 11 gêneros (12 SNPs). Cada SNP individual apresentou validade satisfatória (variando de 29,35 a 88,43, todos F > 10; Tabela 2). A Tabela S3 apresenta detalhes essenciais das VIs.

### Análise de randomização mendeliana no nível de significância de todo o lócus

**Figura 1:**
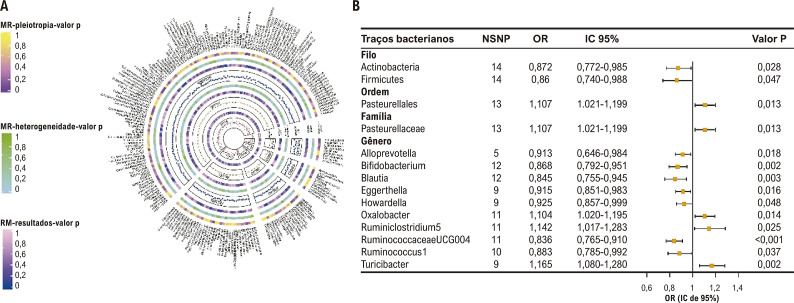
– Análise causal da microbiota intestinal (MI) e fibrilação atrial (FA) no nível de significância de todo o lócus (p<1x10-5). (A) Todos os resultados da análise de randomização mendeliana (RM) e análise de sensibilidade entre MI e FA. (B) Resultados de RM dos táxons da MI com relação causal com a FA usando o método de ponderação de variância inversa (IVW).

Com base no nível de significância de todo o lócus, a Figura 1A descreve graficamente a relação entre MI e FA pela análise de RM. Além disso, uma visão geral abrangente dos resultados pode ser encontrada na Tabela S4. Entre os resultados de RM, descobrimos que a abundância relativa geneticamente prevista de dois filos, uma ordem, uma família e 10 gêneros estava causalmente associada à FA. Em relação às classificações de filos biológicos, o método IVW demonstrou que *Actinobacteria* e *Firmicutes* foram negativamente correlacionados com o risco de FA (Figura 1B). Além disso, as estimativas do IVW por RM indicaram que a ordem de *Pasteurellales* e a família de *Pasteurellaceae* estavam positivamente correlacionadas com o risco de FA (Figura 1B). Em relação ao gênero, as estimativas do IVW por RM indicaram que *Alloprevotella, Bifidobacterium, Blautia, Eggerthella, Howardella, Ruminococcaceae UCG004* e *Ruminococcus1* foram fatores de proteção para FA. Em contraste, *Oxalobacter, Ruminiclostridium5* e *Turicibacter* foram fatores de risco para FA (Figura 1B). Os outros dois táxons da MI (espécie e classe) não mostraram relação com FA por IVW.

A Tabela S5 demonstra ainda os resultados da análise de sensibilidade, que indicam que não houve heterogeneidade ou pleiotropia horizontal em *Pasteurellaceae, Alloprevotella, Bifidobacterium, Eggerthella, Ruminiclostridium5, Ruminococcaceae UCG004, Ruminococcus1, Turicibacter* ou *Pasteurellales*. Além disso, MR-PRESSO não encontrou valores atípicos nesses táxons da MI. Entretanto, a heterogeneidade apareceu em três táxons da MI: *Howardella, Oxalobacter* e *Firmicutes* (Tabela S5). Portanto, usamos os resultados dos efeitos aleatórios do IVW para resolver esse problema. Simultaneamente, um táxon da MI exibiu evidências de pleiotropia horizontal, a saber, *Actinobacteria*, mas a análise MR-PRESSO subsequente indicou que esse táxon em particular não continha nenhum SNP atípico que exigisse remoção (Tabela S5).

### Análise de randomização mendeliana no nível de significância de todo o genoma

**Figura 2:**
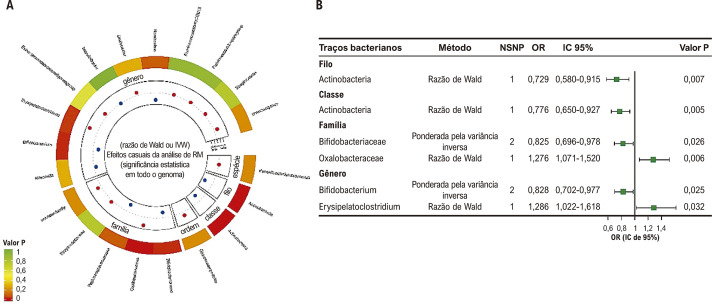
– Análise causal da microbiota intestinal (MI) e fibrilação atrial (FA) no limiar de significância estatística de todo o genoma (p<5x10-8). (A) Todos os resultados da análise de randomização mendeliana (RM) e análise de sensibilidade entre MI e FA. (B) Resultados de RM dos táxons da MI com relação causal com a FA usando o método de ponderação de variância inversa (IVW) ou razão de Wald.

Dependendo do limite de significância estatística de todo o genoma, a Figura 2A representa visualmente a associação entre MI e FA por meio da análise de RM. Além disso, uma apresentação abrangente dos resultados é fornecida na Tabela S6. Entre os resultados de RM, descobrimos que a abundância relativa geneticamente prevista de um filo, uma classe, uma ordem, duas famílias e dois gêneros estava causalmente associada à FA. Os resultados da razão de Wald demonstraram que o filo de *Actinobacteria* e a classe de *Actinobacteria* estavam negativamente correlacionados com o risco de FA (Figura 2B). Quanto à família, *Bifidobacteriaceae* foi negativamente correlacionada com o risco de FA usando o método IVW, e *Oxalobacteraceae* foi positivamente correlacionada com o risco de FA usando o método da razão de Wald (Figura 2B). Além disso, quanto ao gênero, *Bifidobacterium* foi um fator de proteção para FA usando o método IVW, e *Erysipelatoclostridium* foi um fator de risco para FA usando o método da razão de Wald (Figura 2B).

A Tabela S7 exibe os resultados do teste de heterogeneidade conduzido para táxons da MI apresentando dois SNPs. A heterogeneidade e a pleiotropia horizontal não puderam ser examinadas porque apenas um SNP foi incluído nos outros táxons da MI.

## Discussão

Pesquisas anteriores demonstraram o envolvimento significativo da ecologia microbiana intestinal no início e no avanço de várias doenças cardiovasculares, incluindo hipertensão, aterosclerose e insuficiência cardíaca. Li et al.^5^ revelaram que indivíduos com pré-hipertensão e hipertensão apresentam não apenas diminuição da abundância genética e da diversidade α em comparação com controles saudáveis, como também uma proporção elevada de bactérias *Prevotella*. Concomitantemente, uma análise macrogenômica^25^ demonstrou que a MI de indivíduos com doença cardiovascular aterosclerótica apresentava níveis elevados de espécies de *Streptococcus* e *Enterobacteriaceae*, em contraste com a MI de indivíduos saudáveis. Além disso, uma investigação comparativa de bactérias fecais^26^ entre indivíduos com insuficiência cardíaca crônica e seus pares saudáveis indicou que aqueles com insuficiência cardíaca exibiram uma maior colonização de bactérias patogênicas, incluindo *Campylobacter, Shigella, Salmonella* e *Yersinia enterocolitica*.

A FA é um tipo mais específico de DCV. Vários estudos anteriores identificaram perfis de MI distintos entre indivíduos com FA, sendo que a FA demonstra alterações significativas na diversidade microbiana intestinal em comparação com indivíduos controle. Um aumento na abundância de *Ruminococcus, Streptococcus* e *Enterococcus*, juntamente com uma diminuição de *Faecalibacterium, Alistipes, Oscillibacter* e *Bilophila*, foi observado em pacientes com FA.^27^ Além disso, a interrupção do equilíbrio ecológico intestinal tem sido associada ao avanço e à duração da FA. Uma investigação recente de Zuo et al.^28^ revelou distinções na diversidade microbiana e na composição de metabólitos entre pacientes com FA paroxística, com FA persistente e sem FA. Além disso, bactérias específicas exibiram enriquecimentos variados correspondentes a diferentes durações de FA. Por exemplo, na FA persistente, a abundância de *Butyricoccus* e *Paraprevotella* diminuiu, enquanto a abundância de *Dorea* e *Coprococcus* apresentou aumento.^29^ Curiosamente, um aumento no enriquecimento de bactérias benéficas e uma diminuição no enriquecimento de bactérias patogênicas na MI, bem como alterações correspondentes nos níveis de metabólitos, puderam ser observados após a ablação, em comparação com pacientes com FA antes da ablação por radiofrequência.^30^

Entretanto, a maioria dos estudos anteriores foram estudos observacionais e de pequenas coortes, e os tipos de MI incluídos foram frequentemente limitados. Além disso, muitos estudos exploraram apenas as características da MI na FA sem examinar se a MI poderia influenciar o início da FA. Existia, portanto, a necessidade de caracterizar de forma mais completa a relação causal entre MI e FA. Utilizamos os mais amplos e atualizados dados GWAS para MI e FA, para SNPs intimamente relacionados como VIs. Primeiro, por meio da análise de RM no nível de significância de todo o lócus, identificamos *Actinobacteria, Firmicutes, Alloprevotella, Bifidobacterium, Blautia, Eggerthella, Howardella, Ruminococcaceae UCG004* e *Ruminococcus* como sendo negativamente correlacionados com a ocorrência de FA, e *Pasteurellales, Pasteurellaceae, Oxalobacter, Ruminiclostridium5* e *Turicibacter* como sendo positivamente correlacionados com FA. Em segundo lugar, de acordo com o limiar de significância estatística de todo o genoma, identificamos *Actinobacteria, Bifidobacteriaceae* e *Bifidobacterium* como fatores de proteção para o risco de ocorrência de FA, enquanto *Oxalobacteraceae* e *Erysipelatoclostridium* foram fatores de risco para FA. Os resultados para *Actinobacteria, Howardella, Oxalobacter* e *Firmicutes* devem ser interpretados com mais cautela devido à presença de heterogeneidade ou pleiotropia horizontal.

Nosso estudo identificou um total de 10 táxons da MI associados positivamente à FA e sete que foram associados negativamente. Primeiro, *Actinobacteria* e *Bifidobacterium* foram particularmente importantes para reduzir a ocorrência de FA, pois foi comprovado que estavam fortemente correlacionadas negativamente com FA, não apenas no nível de significância de todo o foco, mas também no limiar de todo o genoma, indicando novamente a importância desses dois táxons da MI para prevenir o surgimento de FA. O estudo FINRISK 2002^31^ observou que *Bifidobacterium* estava negativamente correlacionado com a prevalência de FA e positivamente correlacionado a incidentes de FA. Além disso, Li et al.^32^ exibiram uma diminuição significativa na abundância de *Bifidobacterium* em pacientes com FA em comparação aos controles, independentemente de terem recebido terapia anticoagulante oral (ACO) de longo prazo. Apesar da falta de estudos relevantes demonstrando o envolvimento de *Actinobacteria* com o desenvolvimento de FA, Li et al.^32^ também descobriram que as cepas enriquecidas (*Actinobacteria*) presentes em FA submetidas a ACOs de longo prazo estavam positivamente correlacionadas com o tempo de protrombina (p < 0,05), o que pode aumentar o risco de sangramento em pacientes com FA. Notavelmente, a capacidade das *Actinobacteria* de mitigar o risco de desenvolver FA tem sido uma observação única até o momento. Em segundo lugar, embora não tenha mostrado uma associação causal com FA no limiar de significância estatística de todo o genoma, um subconjunto adicional de táxons da MI ainda sugeria o desenvolvimento da FA, incluindo *Firmicutes, Alloprevotella, RuminococcaceaeUCG004*, *Ruminococcus1, Oxalobacter* e *Turicibacter*. Semelhante às nossas descobertas, *Ruminococcus* demonstrou estar negativamente associado às pontuações CHA2DS2-VASc, o que significa que a disponibilidade de *Ruminococcus* pode resultar em um risco reduzido de FA. No entanto, Zuo et al.^27^ detectaram crescimento excessivo de *Ruminococcus* em pacientes com FA. Enquanto isso, outro estudo da mesma equipe^33^ descobriu maior indução de FA em camundongos privados de sono (SD) e descobriu que tais camundongos tinham maiores abundâncias de *Ruminococcus* e *Alloprevotella*, o que sugere que essas espécies de bactérias podem ser alvos promissores para a suscetibilidade à FA mediada pela privação de sono. Também diferiu de nossas descobertas, pois o estudo FINRISK 2002^31^ considerou que *Turicibacter* estava negativamente associado à FA prevalente, enquanto *Firmicutes* foi considerado equivalente em pacientes com FA e controles no estudo de Li et al.^32^ Embora o gênero *Oxalobacter* previsto geneticamente tenha sido positivamente associado ao risco de desenvolver doença arterial coronária em uma análise de RM $(OR)=1,06;p=1,67\times 10^{-4})$ ,^34^ nosso estudo concluiu que *Oxalobacter* e *Oxalobacteraceae* foram fatores de risco para o desenvolvimento de FA, o que complementa e refina as conclusões de estudos anteriores. Por fim, existe um subconjunto de táxons da MI cuja relação com a FA não foi abordada em estudos anteriores, incluindo *Blautia, Eggerthella, Howardella, Pasteurellales, Pasteurellaceae, Ruminiclostridium5* e *Erysipelatoclostridium*. Portanto, nossa pesquisa preenche, de modo satisfatório, essa parte da lacuna.

Os mecanismos que desregulam a ecologia intestinal e, portanto, promovem a FA vêm de duas fontes principais. Primeiro, a desregulação da MI pode causar FA por meio de inflamação. Zhang et al.^35^ demonstraram pela primeira vez que alterações na MI relacionadas à idade levaram ao aumento das concentrações de lipopolissacarídeos (LPS) e à diminuição da tolerância à glicose, o que aumentou a fibrose atrial e promoveu o desenvolvimento de FA. Os mecanismos subjacentes aos efeitos proarrítmicos atriais causados pelos LPS podem ser atribuídos à ligação de nucleotídeos nos átrios e à ativação de vesículas inflamatórias NLRP3. Sequencialmente, os metabólitos derivados da MI também tiveram uma função vital na patogênese da FA. Ao serem absorvidos pelo intestino do hospedeiro, os metabólitos derivados da MI foram capazes de influenciar células imunes no intestino, além de atuar como moléculas de sinalização e influenciar importantes vias metabólicas. Por exemplo, TMAO, que é derivado da colina e da carnitina da dieta, exacerbou o aumento da atividade neural e induziu FA por meio da estimulação atrial, possivelmente porque TMAO poderia estimular a liberação de fatores inflamatórios e ativar a via de sinalização p65 NF-κB.^36^ Além disso, o indoxil sulfato (IS) foi a toxina urêmica mais comum, derivada do metabolismo do triptofano da dieta. Em estudos com animais, o IS pode contribuir para o início da FA ao aumentar a expressão de moléculas de sinalização, como fatores pró-inflamatórios e pró-fibróticos, induzindo assim o estresse oxidativo.^37^ Mais pesquisas básicas ainda são necessárias para explorar se existem mecanismos adicionais pelos quais a MI regula a FA.

Várias imitações desta obra também devem ser abordadas. Primeiro, apesar das três premissas fundamentais da RM terem sido atendidas, nenhuma garantia absoluta de viés instrumental fraco pode ser feita. Em segundo lugar, devido à disponibilidade inadequada de um número substancial de VIs para análise de RM reversa, não conseguimos determinar uma possível relação causal recíproca entre MI e FA. Terceiro, como o GWAS abrangeu apenas populações europeias, as descobertas deste estudo podem não ser universalmente aplicáveis a outros grupos étnicos. Quarto, empregar inúmeras correções estatísticas rigorosas e conservadoras poderia ser excessivamente restritivo, possivelmente negligenciando os táxons da MI que poderiam ter conexões causais com a FA. Considerando a plausibilidade biológica, consequentemente, não incorporamos os resultados de múltiplos testes. Por fim, este estudo marcou a tentativa inaugural de examinar a associação entre táxons da MI e risco de FA por meio de análise de RM no nível de espécie. Entretanto, é importante ressaltar que não foram detectados resultados positivos em nível de espécie. Considerando as distintas fontes de dados utilizadas para a análise em nível de espécie, em contraste com os outros cinco níveis e reconhecendo a diferença substancial nos tamanhos de amostra entre o GWAS conduzido por Goodrich et al.^9^ e o de Kurilshikov et al.,^8^ tornou-se evidente que a seleção potencial de VIs foi bastante limitada. Portanto, essa descoberta no nível de espécie foi apenas uma exploração preliminar.

## Conclusões

Concluindo, este estudo de RM demonstrou o efeito causal da MI na FA, encontrando 10 táxons da MI que foram correlacionados positivamente com a FA, bem como sete que foram correlacionados negativamente. Esses tipos de táxons da MI podem servir como novos biomarcadores e fornecer insights sobre o tratamento e a prevenção da FA.
