# Supplementary material for: A Relação Causal entre Microbiota Intestinal e Fibrilação Atrial: Um Estudo de Randomização Mendeliana de Duas Amostras
Source: Arq Bras Cardiol. 2024 Nov 18;121(11):e20240357. [Article in Portuguese] doi: 10.36660/abc.20240357 (PMC11634295; doi:10.36660/abc.20240357)
Supplement: Translation

# Translation: The Causal Relationship between Gut Microbiota and Atrial Fibrillation: A Two-Sample Mendelian Randomization Study

**DOI:** 10.36660/abc.20240357i

**Authors:** Yuan Zhou, Xuan Wang, Jiongchao Guo, Lei Zhang, Huangsheng Zheng

**Affiliations:** 1 China Department of Cardiology, The First People’s Hospital of Hefei, The Third Affiliated Hospital of Anhui Medical University, Hefei – China; 2 China Department of Health Medicine, The First Affiliated Hospital of Xi’an Jiaotong University, Shanxi Province – China

**Keywords:** Mendelian Randomization Analysis, Gastrointestinal Microbiome, Atrial Fibrillation, Cardiovascular Diseases

## Abstract

**Background:**

Previous studies have adequately characterized the gut microbiota (GM) in atrial fibrillation (AF). Nevertheless, the precise causality between GM and AF remains elusive.

**Objectives:**

This study utilized public data from genome-wide association studies to explore the causality between GM and AF.

**Methods:**

In the first of two rounds of Mendelian randomization (MR) analysis, the instrumental variables (IVs) comprised single nucleotide polymorphisms (SNPs) that fell below the genome-wide statistical significance threshold (5 × 10^-8^). To attain a more comprehensive and inclusive conclusion, we further selected SNPs falling below the locus-wide significance level (1 × 10^-5^) as IVs for the second group. The MR analysis considered the statistically significant causal effect between the specific GM and AF when p < 0.05. Furthermore, in sensitivity analysis, p > 0.05 indicated no heterogeneity and pleiotropy.

**Results:**

At the locus-wide significance threshold, the findings demonstrated a causal impact of GM on AF risk. The inverse variance weighting method indicated that *Actinobacteria*, *Firmicutes*, *Alloprevotella*, *Bifidobacterium*, *Blautia*, *Eggerthella*, *Howardella*, *Ruminococcaceae UCG004*, and *Ruminococcus1* were negatively correlated with AF, while *Pasteurellales*, *Pasteurellaceae*, *Oxalobacter*, *Ruminiclostridium5*, and *Turicibacter* were positively correlated. Furthermore, at the genome-wide significance threshold, *Actinobacteria*, *Bifidobacteriaceae*, and *Bifidobacterium* were protective factors for the risk of developing AF, whereas *Oxalobacteraceae* and *Erysipelatoclostridium* were risk factors for AF. However, sensitivity analyses showed heterogeneity or horizontal pleiotropy within the outcomes for *Actinobacteria*, *Howardella*, *Oxalobacter*, and *Firmicutes*.

**Conclusions:**

This study provides evidence for the existence of both favorable and unfavorable causality of GM on AF risk.

## Introduction

Atrial fibrillation (AF) is a prevalent and intricate arrhythmia that typically manifests in individuals over the age of 60 and currently impacts over 370,000 individuals globally.^1^ AF is distinguished by disruptions in the depolarization process of the atria, potentially resulting in symptoms such as palpitations, chest discomfort, breathlessness, and psychological strain due to irregular atrial contractions.^2^ More serious complications, including ischemic strokes (cerebral infarctions), cardiac insufficiency, and even death, can also occur.^3^ Both clinical and preclinical observations indicate that a multitude of modifiable cardiovascular risk factors coalesce to influence the onset and advancement of AF, such as age, gender, coronary artery disease, heart failure, hypertension, diabetes mellitus, and obesity.^4^ The further elucidation of AF pathogenesis is urgently needed to improve its prevention and treatment.

The human gut serves as a habitat for a diverse array of non-pathogenic microorganisms collectively known as the gut microbiota (GM), which is pivotal in shaping and facilitating the operations of metabolic and immune systems. Recent clinical studies and foundational experiments have revealed that GM potentially influences disease pathways through the metabolites generated within the intestines. Notably, the association between GM and cardiovascular disease (CVD) has gained significant traction in ongoing research. In one study, the fecal contents of hypertensive human donors were transplanted into germ-free mice, whose subsequent direct increases in blood pressure could be attributed to the GM.^5^ Furthermore, the GM was found to contribute to the exacerbation of cardiac function and progression of myocardial fibrosis in mice with heart failure through the metabolism of dietary choline into trimethylamine N-oxide (TMAO).^6^ Although some studies have elaborated upon the diverse GM profile of AF, whether a causal relationship exists between GM and AF remains unclear.

The Mendelian randomization (MR) technique, which involves the amalgamation of aggregated data from genome-wide association studies (GWAS), allows for the mitigation of the confounding influence of factors. Hence, MR stands as a prevalent approach for deducing the existence of a causal connection between exposure and outcome. Genetic variants exhibiting significant associations with exposure are chosen as instrumental variables (IVs) to deduce causality. If exposure is causal, the IVs that affect exposure will influence the outcome proportionally.^7^ In the current study, two-sample MR was performed to examine whether a causal relationship exists between the GM and AF risk.

## Methods

### Data sources

Leveraging data from the MiBioGen consortium, Kurilshikov et al.^8^ harnessed 16S rRNA gene sequencing profiles and genotyping information from a collection of 18,340 samples to explore the intricate interplay between genetic variants and the GM. The entirety of subjects within the MiBioGen consortium encompassed European populations spanning 11 countries, constituting a collective of 25 distinct cohorts. Through an examination of variations in GM taxa, the GWAS study ultimately extracted 122,110 distinct genetic variation sites originating from 211 taxa at various levels (phylum, class, order, family, genus). From this large-scale GWAS, IVs corresponding to GM taxa were derived across five levels. Since the data acquired from the MiBioGen consortium lacked analysis at the species level, we also procured IVs for GM taxa at the species level from the TwinsUK Registry GWAS study.^9^ Goodrich et al. generated IVs of GM taxa at the species level from 1,126 pairs of twins for 16S rRNA sequencing data, ultimately defining four qualifying species.^9^

GWAS summary statistics for AF were excerpted from the newest release (Version R9) of the FinnGen research project (https://r9.finngen.fi/).^10^ Analyses were undertaken using 45,766 AF cases and 191,924 controls after adjusting for age, sex, genetic relatedness, genotyping batch, and the first 10 principal components separately.

In order to design a valid MR study, the required sample size should be calculated. According to asymptotic statistical theory,^11,12^ the minimum sample size derived from the use of available web tools (https://sb452.shinyapps.io/power/) at the given level of significance (< 0.05) and power (> 80%) should be 9900. The sample size included in our research study could fulfill the criteria.

### Single nucleotide polymorphism selection

To ensure the credibility and precision of the conclusions regarding the causal link between GM and AF risk, a series of quality control procedures were employed to choose the most reliable IVs meticulously. First, single nucleotide polymorphisms (SNPs) displaying significant associations with the GM were chosen as IVs, with two distinct thresholds applied to facilitate this selection and achieve a more comprehensive scope of results: a collection of SNPs below the genome-wide statistical significance threshold (5 × 10^-8^), as well as an additional group of SNPs below the locus-wide significance level (1 × 10^-5^).^13^ Second, it was necessary to assess the strength of the correlation between IVs and exposure. Such evaluations are typically accomplished through the F-statistic, which was calculated as $F=R^{2}(n-k-1)/k\left(1-R^{2}\right)$ , where R^2^ represents the exposure variance explained by the selected SNPs, n is the sample size, and k represents the number of included IVs. If the F-statistic is less than 10, it signifies a weak connection between IVs and exposure, so the SNPs that contribute to these IVs were excluded. Second, the threshold for the minor allele frequency (MAF) of the variant under consideration was set at 0.01. Third, SNPs in high linkage disequilibrium (LD) were excluded from the IVs; the presence of substantial LD could potentially introduce biased outcomes. In this study, LD among the included SNPs was assessed by the clumping process (R^2^ < 0.001, and clumping distance = 10,000 kb). Fourth, a pivotal facet of MR involves confirming that the effects of the SNPs on the exposure pertaining to the same allele align with the effects on the outcome. In adherence to this principle, palindromic SNPs were excluded from the IVs. Fifth, in instances where SNPs linked to exposure were not identified in the outcome GWAS, proxy SNPs that displayed significant associations with the pertinent variants were substituted (r^2^ > 0.8).

### The assumptions of mendelian randomization

To perform an accurate and standardized two-sample MR analysis, the following three assumptions had to be met:^14^ (1) the IVs eventually included for utilization must exhibit a strong association with GM taxa; (2) the included IVs and confounders (influencing the GM and AF) must remain independent of each other; and (3) no horizontal pleiotropy should exist, meaning that the IVs affect AF only through GM taxa. Our findings were simultaneously reported following the MR-STROBE guidance^15^ (Table S1). This study did not require informed consent or ethical approval because it used publicly available data.

### Statistical analysis

In the present study, we employed different MR techniques, including inverse variance weighting (IVW), MR-Egger, weighted median, simple mode, and weighted mode, to ascertain the potential causal influence of GM composition on AF risk. In this scenario, the primary method employed for MR analysis was IVW, which essentially functions as a meta-analysis technique that transforms into a weighted regression of the outcome effects of IVs on the exposure effects to yield a comprehensive estimate of the influence of the GM on the risk of AF while constraining the intercept to zero. In the absence of horizontal pleiotropy, IVW could effectively circumvent the influence of confounding factors, allowing for unbiased estimates.^16^ In addition, several other methods exist to complement the IVW results. MR-Egger regression can identify and account for pleiotropy, although this approach often generates estimates with limited precision.^17^ The weighted median approach furnishes reliable estimates under the assumption that a minimum of 50% of the IVs are valid.^18^ While the simple mode may not possess the same level of potency as IVW, it does offer resilience against the effects of multivalency.^19^ Finally, the weighted mode is susceptible to variations in the selection of bandwidth for mode estimation.^20^

Sensitivity analyses were usually performed in three steps. MR-Egger regression, a method that identifies and accounts for pleiotropy in MR analysis while deriving estimates of causal effects and scrutinizing whether the outcomes are influenced by directed horizontal pleiotropy, was initially conducted to evaluate the potential presence of horizontal pleiotropy effects among the included SNPs.^17,21^ Considering the limitations in accuracy and statistical power associated with MR-Egger regression, the MR pleiotropy residual sum and outlier (MR-PRESSO) approach was employed to identify potential outliers that might indicate pleiotropy bias and subsequently correct for horizontal pleiotropy effects.^22^ Finally, Cochran’s Q statistic was employed to measure the degree of heterogeneity among the selected SNPs.^23,24^

All statistical analyses were executed using R software (Version 4.3.1). The R package TwoSampleMR (version 0.5.7, Stephen Burgess, Chicago, IL, USA) was used to perform MR analyses of causality between the GM and AF. p < 0.05 was considered a potentially statistically significant causal effect between exposure and outcome.

## Results

### Data Sources

The Central Figure illustrates the study design and specific flow of MR analysis between the GM and AF. GWAS summary statistics for the GM were obtained from the MiBioGen consortium and the TwinsUK Registry consortium, which contain 18,340 and 1,126 individuals, respectively. Moreover, GWAS summary statistics for AF were obtained from the FinnGen consortium, which contains 45,766 AF cases and 191,924 controls. Table 1 shows the details of three datasets.

**Table 1:** – Description of data sources for gut microbiota and atrial fibrillation

Traits	Consortium	Sample size	Populations	Year	Journal
Gut Microbiota	MiBioGen	18,340 individuals	European	2021	Nature Genetics
	TwinsUK Registry	1,126 individuals	European	2016	Cell Host & Microbe
Fibrilação atrial	FinnGen (R9)	45,766 cases and 191,924 controls	European	2022	Nature

### Selection of Instrumental Variables

After a rigorous quality control step, 2,182 SNPs were identified as IVs associated with 207 GM taxa at the locus-wide significance level (p < 1 × 10^-5^). These taxa comprised four species (14 SNPs), nine phyla (102 SNPs), 10 classes (109 SNPs), 19 orders (210 SNPs), 35 families (385 SNPs), and 130 genera (1,362 SNPs). Each SNP showed sufficient validity (ranging from 14.59–88.43, all F > 10; Table 2). Table S2 provides comprehensive information about the IVs.

**Table 2:** – Selection of IVs after quality control

Taxonomies	p < 1e-05			p < 5e-08		
	Taxa	IVs	Range (F statistics)	Taxa	IVs	Range (F statistics)
Species	4	14	22.50-30.84	1	2	30.25-30.84
Phylum	9	102	16.97-58.16	1	1	58.16
Class	10	109	17.61-85.38	1	1	85.38
Order	19	210	17.68-85.37	1	1	30.07
Family	35	385	16.91-85.37	5	6	29.81-85.37
Genus	130	1362	14.59-88.43	11	12	29.35-88.43
Total	207	2182	14.59-88.43	20	23	29.35-88.43

*IVs: instrumental variables.*

Furthermore, at the genome-wide statistical significance threshold (p < 5 × 10^-8^), only 23 SNPs were identified as IVs, associated with 20 GM taxa. These taxa comprised one species (2 SNPs), one phylum (1 SNP), one class (1 SNP), one order (1 SNP), five families (6 SNPs), and 11 genera (12 SNPs). Every individual SNP exhibited satisfactory validity (ranging from 29.35–88.43, all F > 10; Table 2). Table S3 presents essential details concerning the IVs.

### Mendelian randomization analysis of locus-wide significance level

Based on the locus-wide significance level, Figure 1A graphically depicts the relationship between GM and AF by MR analysis. Additionally, a comprehensive overview of the outcomes can be found in Table S4. Among the MR results, we found the genetically predicted relative abundance of two phyla, one order, one family, and 10 genera was causally associated with AF. Regarding biological phylum classifications, the IVW method demonstrated that *Actinobacteria* and *Firmicutes* were negatively correlated with the risk of AF (Figure 1B). Moreover, the MR estimates of IVW indicated that the order of *Pasteurellales* and the family of *Pasteurellaceae* were positively correlated with AF risk (Figure 1B). Regarding genus, the MR estimates of IVW indicated that *Alloprevotella*, *Bifidobacterium*, *Blautia*, *Eggerthella*, *Howardella*, *Ruminococcaceae UCG004*, and *Ruminococcus1* were protective factors for AF. In contrast, *Oxalobacter*, *Ruminiclostridium5*, and *Turicibacter* were risk factors for AF (Figure 1B). The other two GM taxa (species and class) did not show a relationship with AF by IVW.

**Figure 1:** – Causal analysis of gut microbiota (GM) and atrial fibrillation (AF) at the locus-wide significance level (p<1x10-5). (A) All results of Mendelian randomization (MR) analysis and sensitivity analysis between GM and AF. (B) MR results of GM taxa with a causal relationship to AF using the inverse variance weighting (IVW) method.

Table S5 further demonstrates the results of the sensitivity analysis, which indicate that no heterogeneity or horizontal pleiotropy was present in *Pasteurellaceae*, *Alloprevotella*, *Bifidobacterium*, *Eggerthella*, *Ruminiclostridium5*, *Ruminococcaceae UCG004*, *Ruminococcus1*, *Turicibacter,* or *Pasteurellales*. Furthermore, MR-PRESSO did not find outliers in these GM taxa. However, heterogeneity appeared in three GM taxa: *Howardella*, *Oxalobacter*, and *Firmicutes* (Table S5). We thus used the random effects results of IVW to solve this problem. Simultaneously, one GM taxon exhibited evidence of horizontal pleiotropy, namely *Actinobacteria*, but subsequent MR-PRESSO analysis indicated that this particular taxon did not contain any outlier SNPs requiring removal (Table S5).

### Mendelian randomization analysis of genome-wide significance level

Depending on the genome-wide statistical significance threshold, Figure 2A visually represents the association between GM and AF through MR analysis. Furthermore, a comprehensive presentation of results is provided in Table S6. Among the MR results, we found the genetically predicted relative abundance of one phylum, one class, one order, two families, and two genera was causally associated with AF. The Wald ratio results demonstrated that the phylum of *Actinobacteria* and the class of *Actinobacteria* were negatively correlated with the risk of AF (Figure 2B). As for family, *Bifidobacteriaceae* was negatively correlated with AF risk using the IVW method, and *Oxalobacteraceae* was positively correlated with AF risk using the Wald ratio method (Figure 2B). Moreover, as for genus, *Bifidobacterium* was a protective factor for AF using the IVW method, and *Erysipelatoclostridium* was a risk factor for AF using the Wald ratio method (Figure 2B).

**Figure 2:** – Causal analysis of gut microbiota (GM) and atrial fibrillation (AF) at the genome-wide statistical significance threshold (p<5x10-8). (A) All results of Mendelian randomization (MR) analysis and sensitivity analysis between GM and AF. (B) MR results of GM taxa with a causal relationship to AF using the inverse variance weighting (IVW) or Wald ratio method.

Table S7 displays the outcomes of the heterogeneity test conducted for GM taxa featuring two SNPs. Heterogeneity and horizontal pleiotropy could not be examined because only one SNP was included in the other GM taxa.

## Discussion

Earlier research has demonstrated the significant involvement of gut microbial ecology in the onset and advancement of various CVDs, encompassing conditions such as hypertension, atherosclerosis, and heart failure. Li et al.^5^ revealed that individuals with prehypertension and hypertension exhibit not only diminished gene abundance and α-diversity compared to healthy controls but also an elevated proportion of *Prevotella* bacteria. Concurrently, a macrogenomic analysis^25^ demonstrated that the GM of individuals with atherosclerotic cardiovascular disease harbored elevated levels of *Streptococcus* and *Enterobacteriaceae* species, in contrast to the GM of healthy individuals. Furthermore, a comparative investigation of fecal bacteria^26^ between individuals with chronic heart failure and their healthy counterparts indicated that those with heart failure exhibited higher colonization of pathogenic bacteria, including *Campylobacter*, *Shigella*, *Salmonella*, and *Yersinia enterocolitica*.

AF is a more specific type of CVD. Numerous preceding studies have identified distinct GM profiles among individuals with AF, with AF showing significant alterations in gut microbial diversity compared to control individuals. An increased abundance of *Ruminococcus*, *Streptococcus*, and *Enterococcus*, along with a decrease in *Faecalibacterium*, *Alistipes*, *Oscillibacter*, and *Bilophila*, was observed in patients with AF.^27^ Furthermore, disruption in gut ecological balance has been linked to the advancement and duration of AF. A recent investigation by Zuo et al.^28^ revealed distinctions in microbial diversity and metabolite composition among patients with paroxysmal AF, with persistent AF, and without AF. Moreover, specific bacteria exhibited varying enrichments corresponding to different durations of AF. For instance, in persistent AF, the abundance of *Butyricoccus* and *Paraprevotella* was diminished, while the abundance of *Dorea* and *Coprococcus* showed an increase.^29^ Interestingly, an increase in the enrichment of beneficial bacteria and a decrease in that of pathogenic bacteria in the GM, as well as corresponding changes in metabolite levels, could be observed after ablation, compared to patients with AF before radiofrequency ablation.^30^

However, most previous studies have been observational and small cohort studies, and the types of GM included were often limited. In addition, many studies have merely explored GM characteristics in AF without examining whether GM could influence the onset of AF. A need thus existed to characterize the causal relationship between the GM and AF more fully. We used the largest and most up-to-date GWAS data for GM and AF for closely related SNPs as IVs. First, through MR analysis at the locus-wide significance level, we identified *Actinobacteria*, *Firmicutes*, *Alloprevotella*, *Bifidobacterium*, *Blautia*, *Eggerthella*, *Howardella*, *Ruminococcaceae UCG004*, and *Ruminococcus* as being negatively correlated with the occurrence of AF, and *Pasteurellales*, *Pasteurellaceae*, *Oxalobacter*, *Ruminiclostridium5,* and *Turicibacter* as being positively correlated with AF. Second, under the genome-wide statistical significance threshold, we identified *Actinobacteria*, *Bifidobacteriaceae,* and *Bifidobacterium* as protective factors for the risk of AF occurrence, whereas *Oxalobacteraceae* and *Erysipelatoclostridium* were risk factors for AF. The results for *Actinobacteria*, *Howardella*, *Oxalobacter,* and *Firmicutes* must be interpreted with more caution due to the presence of heterogeneity or horizontal pleiotropy.

Our study identified a total of 10 GM taxa positively associated with AF and seven that were negatively associated. First, *Actinobacteria* and *Bifidobacterium* were particularly important for reducing the occurrence of AF, as they were proved to be markedly negatively correlated with AF not only at the focus-wide significance level but also at the genome-wide threshold, again indicating the importance of these two GM taxa for preventing the emergence of AF. FINRISK 2002 study^31^ noted that *Bifidobacterium* was negatively correlated with AF prevalence and positively collateral to AF incidents. Furthermore, Li et al.^32^ exhibited a significant decrease in *Bifidobacterium* abundance in patients with AF compared to controls, irrespective of whether they received long-term oral anticoagulant (OAC) therapy. Despite the lack of relevant studies demonstrating the involvement of *Actinobacteria* with the development of AF, Li et al.^32^ nonetheless also found that the enriched strains (*Actinobacteria*) present in AF undergoing long-term OACs were positively correlated with prothrombin time (p < 0.05), which may enhance the bleeding risk in patients with AF. Notably, the ability of *Actinobacteria* to mitigate the risk of developing AF has been a unique observation to date. Second, while not showing a causal association with AF at the genome-wide statistical significance threshold, an additional subset of GM taxa were still suggestive of AF development, including *Firmicutes*, *Alloprevotella*, *RuminococcaceaeUCG004*, *Ruminococcus1*, *Oxalobacter,* and *Turicibacter*. Similar to in our findings, *Ruminococcus* was shown to be negatively associated with CHA2DS2-VASc scores, meaning that the availability of *Ruminococcus* may result in a reduced risk of AF. Nonetheless, Zuo et al.^27^ detected *Ruminococcus* overgrowth in AF patients. Meanwhile, another study by the same team^33^ uncovered higher AF induction in sleep-deprived (SD) mice and found that such mice had higher abundances of *Ruminococcus* and *Alloprevotella*, implying that these species of bacteria may be promising targets for SD-mediated AF susceptibility. It also differed from our findings, since the FINRISK 2002 study^31^ considered *Turicibacter* to be negatively linked to prevalent AF, whereas *Firmicutes* were seen to be equivalent in patients with AF and controls in the Li et al. study.^32^ While the genetically predicted genus *Oxalobacter* was positively associated with the risk of developing coronary artery disease in an MR analysis $\text{ [odds ratio }(OR)=1.06;p=1.67\times 10^{-4})$ ,^34^ our study concluded that *Oxalobacter* and *Oxalobacteraceae* were risk factors for developing AF, which complements and refines the conclusions of prior studies. Finally, a subset of GM taxa whose relationship to AF has not been addressed in previous studies exists, including *Blautia*, *Eggerthella*, *Howardella*, *Pasteurellales*, *Pasteurellaceae*, *Ruminiclostridium5*, and *Erysipelatoclostridium*. Our research thus fills this part of the gap satisfactorily.

The mechanisms that dysregulate gut ecology and thus promote AF come from two main sources. First, GM dysregulation could cause AF to occur through inflammation. Zhang et al.^35^ demonstrated for the first time that age-related GM alterations led to increased lipopolysaccharide (LPS) concentrations and impaired glucose tolerance, which enhanced atrial fibrosis and promoted the development of AF. The mechanisms underlying the atrial proarrhythmic effects driven by LPS could be attributed to the binding of nucleotides in the atria and the activation of NLRP3 inflammatory vesicles. Sequentially, GM-derived metabolites also had a vital function in the pathogenesis of AF. By being absorbed into the host intestine, GM-derived metabolites were able to influence immune cells in the gut, as well as act as signaling molecules and influence important metabolic pathways. For example, TMAO, which is derived from dietary choline and carnitine, exacerbated increased neural activity and induced AF via atrial pacing, possibly because TMAO could stimulate the release of inflammatory factors and activate the p65 NF-κB signaling pathway.^36^ In addition, indoxyl sulfate (IS) was the most common uremic toxin derived from the metabolism of dietary tryptophan. In animal studies, IS could contribute to the initiation of AF by upregulating the expression of signaling molecules such as pro-inflammatory and pro-fibrotic factors, thereby inducing oxidative stress.^37^ Further basic research is still needed to explore whether additional mechanisms exist by which the GM regulates AF.

Several imitations of this work must also be addressed. First, despite the three fundamental assumptions of MR being met, no absolute assurance of weak instrumental bias could be made. Second, owing to the inadequate availability of a substantial number of IVs for reverse MR analysis, we were unable to ascertain a potential reciprocal causal relationship between GM and AF. Third, since the GWAS encompassed solely European populations, the findings of this study might not be universally applicable to other ethnic groups. Fourth, employing numerous stringent and conservative statistical corrections could potentially be excessively restrictive, possibly neglecting the GM taxa that could have causal connections to AF. Considering biological plausibility, we consequently refrained from incorporating the results of multiple tests. Lastly, this study marked the inaugural attempt to scrutinize the association between GM taxa and AF risk via MR analysis at the species level. However, it is noteworthy that positive outcomes at the species level were not detected. Considering the distinct data sources utilized for the species-level analysis, in contrast to the other five levels and recognizing the substantial difference in sample sizes between the GWAS conducted by Goodrich et al.^9^ and that of Kurilshikov et al.,^8^ it became evident that the potential selection of IVs was greatly constrained. Thus, this discovery at the species level was only a preliminary exploration.

## Conclusions

In conclusion, this MR study demonstrated the causal effect of GM on AF, finding 10 GM taxa that were positively correlated with AF, as well as seven that were negatively correlated. These types of GM taxa may serve as new biomarkers and provide insights into the treatment and prevention of AF.
